# The REPERFUSE study protocol: The effects of vasopressor therapy on renal perfusion in patients with septic shock—A mechanistically focused randomised control trial

**DOI:** 10.1371/journal.pone.0304227

**Published:** 2024-06-13

**Authors:** Rory McDonald, James Watchorn, Reena Mehta, Marlies Ostermann, Sam Hutchings

**Affiliations:** 1 Department of Inflammation Biology, School of Immunology and Microbial Sciences, King’s College London, London, United Kingdom; 2 Academic Department of Anaesthesia and Critical Care, Royal Centre for Defence Medicine, Birmingham, United Kingdom; 3 Department of Critical Care, King’s College Hospital, London, United Kingdom; 4 Pharmacy Department, King’s College Hospital, London, United Kingdom; 5 School of Cancer and Pharmaceutical Sciences, King’s College London, London, United Kingdom; 6 Department of Critical Care, Guy’s & St Thomas’ Hospital, London, United Kingdom; CHU Nantes, FRANCE

## Abstract

**Introduction:**

Acute kidney injury (AKI) is a common complication of septic shock and together these conditions carry a high mortality risk. In septic patients who develop severe AKI, renal cortical perfusion is deficient despite normal macrovascular organ blood flow. This intra-renal perfusion abnormality may be amenable to pharmacological manipulation, which may offer mechanistic insight into the pathophysiology of septic AKI. The aim of the current study is to investigate the effects of vasopressin and angiotensin II on renal microcirculatory perfusion in a cohort of patients with septic shock.

**Methods and analysis:**

In this single centre, mechanistically focussed, randomised controlled study, 45 patients with septic shock will be randomly allocated to either of the study vasopressors (vasopressin or angiotensin II) or standard therapy (norepinephrine). Infusions will be titrated to maintain a mean arterial pressure (MAP) target set by the attending clinician. Renal microcirculatory assessment will be performed for the cortex and medulla using contrast-enhanced ultrasound (CEUS) and urinary oxygen tension (pO2), respectively. Renal macrovascular flow will be assessed via renal artery ultrasound. Measurement of systemic macrovascular flow will be performed through transthoracic echocardiography (TTE) and microvascular flow via sublingual incident dark field (IDF) video microscopy. Measures will be taken at baseline, +1 and +24hrs following infusion of the study drug commencing. Blood and urine samples will also be collected at the measurement time points. Longitudinal data will be compared between groups and over time.

**Discussion:**

Vasopressors are integral to the management of patients with septic shock. This study aims to further understanding of the relationship between this therapy, renal perfusion and the development of AKI. In addition, using CEUS and urinary pO2, we hope to build a more complete picture of renal perfusion in septic shock by interrogation of the constituent parts of the kidney. Results will be published in peer-reviewed journals and presented at academic meetings.

**Trial registration:**

The REPERFUSE study was registered on Clinical Trials.gov (NCT06234592) on the 30^th^ Jan 24.

## Introduction

Septic shock, whereby a vasopressor agent is required to maintain organ perfusion, is the leading cause of acute kidney injury (AKI) in the critically ill [1]. The mortality rate associated with septic shock is in excess of 40% [2]. However, in combination with AKI, this increases up to 70% [3]. Prevention of AKI is therefore of vital importance. However, attempts to do so are limited by a poor understanding of the pathophysiology of this condition and the critical limitations of current diagnostic and renal monitoring techniques.

Acute kidney injury in the setting of shock was historically considered a result of systemic factors leading to a reduction in global blood flow and renal perfusion, with resultant ischaemic tissue injury. However, septic AKI is now thought to result from the complex interplay of many processes including: alterations in perfusion, inflammation, changes in cellular processes as well as iatrogenic variables [3–5].

Animal models examining renal perfusion in septic shock have generated conflicting results. However, once a hyperdynamic state is established, renal blood flow is consistently found to be preserved or increased [6]. In an ovine hyperdynamic septic shock series, a well studied large animal model thought more reflective of human septic shock, renal functional impairment is repeatedly seen despite preserved or increased organ perfusion [7–9]. Altered flow within the kidney itself may therefore contribute to the development of septic AKI. However, clinical studies of renal blood flow and cortical perfusion in septic shock are limited because of the difficulties in achieving reliable observations. Renal blood flow has been measured as unchanged or reduced via ultrasound Doppler, thermodilution or magnetic resonance imaging [10–12]. These different findings may reflect limitations in the individual studies, or the multiple variables that distinguish human septic shock from experimental models.

Until recently, non-invasive measurement of the microcirculation within the kidney has posed a challenge. However, the emerging technique of contrast enhanced ultrasound (CEUS) now allows bedside assessment of renal cortical microvascular blood flow in the critically ill. In clinical studies, CEUS has demonstrated impaired renal cortical perfusion in septic AKI [10, 13, 14]. In addition, recent work by Watchorn *et al* demonstrated impaired renal cortical perfusion in patients with septic shock who developed severe AKI compared to those with either mild injury or preserved renal function. This occurred despite no difference in renal blood flow and measures of systemic macro- or sublingual microcirculation [10]. These results again raise the possibility of intra-renal processes contributing to the development of septic AKI, and therefore the possibility of therapeutic manipulation.

Vasopressin and angiotensin II are two agents that have generated interest in relation to AKI due to renal specific pharmacokinetics [15]. Vasopressin is routinely used in clinical practice and is a recommended second line vasopressor in refractory septic shock [16]. Angiotensin II is the latest vasopressor studied in septic shock. In the ATHOS-3 trial, it was shown to be both safe and effective in the management of shocked critically ill patients [17].

Urinary oxygen tension (pO2) has been proposed as a proxy measure for medullary oxygenation, a tissue difficult to study by other methods. This monitoring modality may give information on the balance between microcirculatory perfusion and oxygen consumption in this tissue [18]. In the clinical setting, the utility of urinary pO2 and its association with AKI has mainly been studied in cardiac surgery requiring cardiopulmonary bypass [19–21]. However, its potential in septic shock is yet to be fully evaluated.

An expert panel has reviewed the data on sepsis-associated AKI (SA-AKI) and identified key areas for urgent research [5]:

1. What is the utility of haemodynamic monitoring in SA-AKI?
2. What is the role of the assessment of renal perfusion using ultrasound, and assessment of mean perfusion pressure in managing SA-AKI?
3. Does the choice of vasopressor agent affect the course of SA-AKI?

This trial aims to further mechanistic understanding of AKI associated with septic shock and explore the relationship between vasopressor use and systemic and renal perfusion, both macro- and microvascular. In addition, we aim to evaluate the utility of urinary oxygen tension monitoring in critically ill patients with septic shock at risk of AKI.

## Materials and methods

### Aim

The primary objective of the study is to assess the effect of differential vasopressor therapies (norepinephrine, vasopressin and angiotensin II) on renal cortical perfusion in patients with septic shock. The primary outcome measure is the absolute CEUS derived cortical mean transit time (mTT) at the +24hr timepoint. Secondary objectives are to assess the temporal effects of study vasopressor on renal cortical perfusion at other study time points, the relationship between continuous urinary oximetry and renal cortical perfusion, and the relationship between new kidney biomarkers and other measures at the +24hr timepoint. Exploratory objectives include: the temporal effects on IDF video microscopy measures of microcirculatory flow between treatment groups, and the relation of biomarkers for inflammation and endothelial activation to treatment group and other study measures.

### Trial design

The REPERFUSE study is a single centre, mechanistically focussed, randomised controlled trial. Full trial registration data is outlined in S1 File. Following randomisation, baseline measurements will be made using the described devices: CEUS, abdominal ultrasound, IDF video microscopy and transthoracic echocardiography (TTE). In addition, a urinary oximetry probe will be inserted to allow continuous measurement of urinary pO2 for the duration of the study. Study drug, or standard care vasopressor, will be introduced or titrated in a protocolised manner in order to achieve a target MAP. Individual participant MAP targets will be set by the attending clinician and maintained for the duration of the study. During the study window, serial or continuous measures, as listed above, will be made alongside collection of participant clinical and physiological data. In addition, blood and urine samples will be collected for biomarker analysis. All procedures will be undertaken by an intensivist specifically trained in the individual techniques.

### Study setting

Critically unwell patients with septic shock are a challenging cohort to recruit, particularly when observations are required early following admission to critical care. In order to make recruitment feasible, this study requires a setting with a high incidence of potentially suitable patients. The Department of Critical Care at King’s College Hospital, London, UK, is a tertiary unit with suitable clinical workload. In addition, and subject to approvals, a second tertiary critical care unit will be used to support successful participant recruitment.

### Sample size

A power calculation based on detecting a difference in CEUS derived mTT of 5±2s (based on differences between groups with severe and mild AKI in the Microshock-Renal study [10]) and assuming a power of 0.8 and alpha of 0.05 produces a sample size of 12 per group, or 36 participants in total. Accounting for drop-outs, we will aim to recruit 15 patients per group to give a total sample size of 45.

### Inclusion and exclusion criteria

For inclusion into the study, patients must meet the following criteria, based on the Third International Consensus Definitions for Sepsis and Septic Shock [2].

- Age >18 years
- Within 48hrs of intensive care admission
- Evidence of suspected or confirmed infection
- Sequential Organ Failure (SOFA) score increase of 2 or more (assuming a baseline of 0 if no previous measures)
- Administration of norepinephrine infusion as the sole vasopressor agent in a dose of >0.1mcg/kg/min
- Lactate >2mmol/L at any stage prior to randomisation

Exclusion criteria are based on intolerance or contraindication to ultrasound contrast or study vasopressors.

- Known intolerance to Sonovue^™^ contrast medium, vasopressin or angiotensin II
- Patients receiving other vasopressor agents in addition to norepinephrine
- Patients with known chronic kidney disease (CKD) stage 4 or 5 (baseline glomerular filtration rate (GFR) <30mls/min) or a renal transplant
- Patients receiving extra corporal membrane oxygenation (ECMO)
- Patients with acute occlusive coronary syndromes requiring intervention
- Patients with mesenteric ischaemia
- Patients with a history or presence of aortic dissection or abdominal aortic aneurysm
- Patients with Raynaud’s syndrome or acute vaso-occlusive conditions
- Pregnancy
- Patients with an expected lifespan <24hrs in whom the primary treatment intent is palliative

### Patient screening, recruitment, randomisation and blinding

All new admissions will be screened by research staff to assess eligibility using the clinical information system. Patients identified as potentially eligible will be recorded in a screening log and the attending consultant approached to provide an opinion on suitability based on the inclusion and exclusion criteria.

Patients with septic shock, eligible for inclusion in this study, are critically unwell and often receiving sedative medications. They typically lack capacity to provide informed consent. Once an eligible patient is identified for the trial, a registered medical practitioner independent of the study will be asked whether the patient is suitable for enrolment. This medical practitioner will usually be the patient’s attending consultant. If the patient is deemed suitable they will be enrolled into the study. This method of ‘deferred consent’ is covered by an emergency waiver of consent under the UK Mental Capacity Act UK (2019) approved by London South East Research Ethics Committee (REC).

Within a reasonable time frame after study enrolment, a study investigator will approach a Personal Consultee, typically a relative or close friend. The Personal Consultee will be asked to give an opinion on the patient’s likely wishes regarding participating in research. If their opinion is that the patient would not choose to participate in research, then study intervention will cease, including transitioning the patient back to standard vasopressor therapy if applicable, and no further data collection will occur. In the event that there is no Personal Consultee available a Nominated Consultee, an independent clinician not associated with the conduct of the study, will be used.

Informed deferred written consent will occur once patients are deemed to have regained full capacity. If consent is not granted no further data collection will occur. In this scenario patients will be asked whether stored samples can be kept and analysed, and any data that has already been collected used. If consent is not granted for this all samples and data already obtained will be destroyed. The patient’s decision will be final and supersede any Personal or Nominated Consultee. Where a patient dies before they regain capacity to consent all samples and data will be retained.

Treatment assignment will be through random allocation to one of three groups using random permuted block allocation with a block size of 6. Sealed Envelope^™^ software will be used to allocate patients to groups. The investigators enrolling participants have had no involvement the sequence generation. Groups will be:

- Norepinephrine alone (standard care)
- Norepinephrine + vasopressin
- Norepinephrine + angiotensin II

Given the nature of the study it will not be possible to blind either investigators performing study measurements or care providers. However, data collected will be coded and filed such that offline data analysis is performed blinded to group allocation.

### Interventions

**Fig 1:**
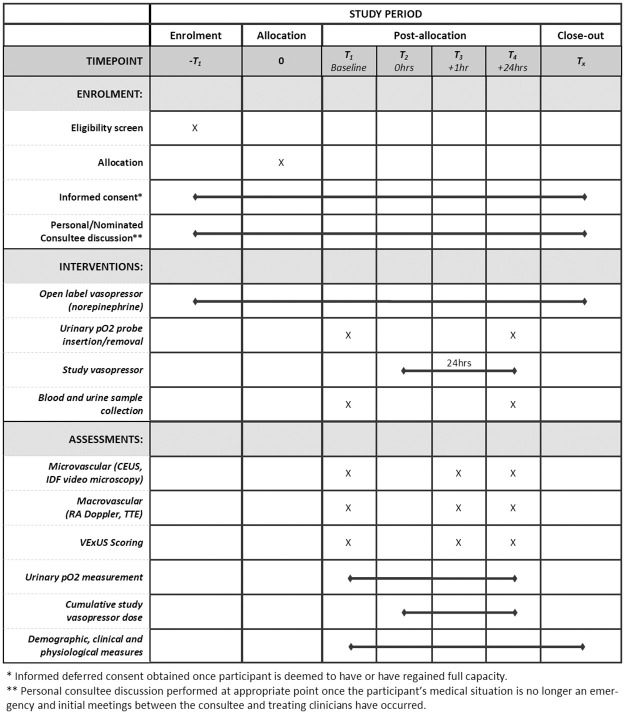
SPIRIT schedule of enrolment, interventions and assessments. CEUS = contrast enhanced ultrasound, IDF = incident dark field, RA = renal artery, TTE = transthoracic echocardiography, VExUS = venous excess ultrasound. X = specified observation.

The SPIRIT schedule of study enrolment, interventions and assessments is outlined in Fig 1. For SPIRIT checklist, see S2 File. Following participant enrolment and study arm allocation, baseline data collection using the subject devices will be undertaken in addition to collection of blood and urine samples, timepoint 1 (T1). On completion of baseline measurements, participants randomised to receive either vasopressin or angiotensin II will have infusions of these drugs commenced, timepoint 2 (T2). Study vasopressors will be titrated in a protocolised manner until the maximum dose of 0.04 IU/min vasopressin or 40 ng/kg/min of angiotensin II is achieved, or the target MAP is reached. As these agents are commenced, norepinephrine dosing will be reduced to maintain the target MAP directed by the attending clinician. If the target MAP is achieved with the study vasopressor alone then norepinephrine will be discontinued. If, despite cessation of norepinephrine, the MAP target is exceeded then the study vasopressor will be weaned in a protocolised manner. The protocol for escalating and weaning vasopressors is outlined in S3 File. Data collection using the subject devices will be repeated one hour after steady state is achieved in terms of haemodynamics and study vasopressor dose, timepoint 3 (T3). Repeat data collection will occur and further blood and urine samples taken 24 hours later, timepoint 4 (T4). All procedures and assessments will be undertaken by a single intensivist specifically trained in the individual techniques. On study completion, vasopressin or angiotensin II will be gradually reduced and norepinephrine increased to reflect standard care by the clinical team.

#### Renal contrast enhanced ultrasound: Haemodynamic assessment of the renal cortical microcirculation

Renal ultrasound and contrast enhanced ultrasound (CEUS) will be performed using an Affiniti Ultrasound System (Philips, UK).

Conventional grayscale ultrasound imaging will be performed. Where satisfactory ultrasound windows cannot be obtained this will be recorded and the patient excluded. The investigator will vary the pulse repetition frequency, focal zone, gain, and wall filter as necessary to obtain optimal sonograms in each case. Both kidneys will be visualized and the most accessible chosen to perform the study. Baseline grayscale and colour Doppler sonographic images will be recorded. A low–mechanical index technique (range: 0.04–0.1) for CEUS will be utilised. A continuous infusion of 4.8 mL of SonoVue^™^ (Bracco SpA, Italy) contrast agent will be administered at a rate of 1 ml/min.

Images of the entire examination will be digitally recorded. After 2 minutes infusion time steady state will be achieved and 5 high frequency ultrasound pulses will be delivered, one every 30 seconds. Destruction-replenishment kinetics will be quantified by post-processing, performed offline using VueBox^™^ (Bracco Diagnostic Imaging, Switzerland). Cortical regions lying in proximity to the probe with good views and reliable visible reperfusion will be identified as regions of interest and the following variables calculated: cortical mean transit time (mTT), perfusion index (PI), wash-in rate (WiR) and relative blood volume (RBV).

#### Incident dark field video microscopy: Haemodynamic assessment of the systemic microcirculation

Videos of the sublingual microcirculation will be acquired using an Incident Dark Field (IDF) video-microscope (Cytocam, Braedius Medical, The Netherlands).

Following suctioning of the oropharynx, a gauze swab will be applied to gently remove saliva from the mucosal surface. The camera probe will be applied to the sublingual area and images selected, taking care to exclude areas of buccal microcirculation with large numbers of looped vessels. The device will be focussed until individual erythrocytes can be visualized within capillaries and the brightness setting adjusted to produce an acceptable degree of contrast between blood vessels and background tissue. At all times, pressure artefact will be scrupulously avoided by applying only the minimal amount of pressure necessary to obtain an image. Stopped or reversed flow in larger venules will be taken as a sign of pressure artefact, necessitating adjustment of the camera or selection of a different area of the microcirculation for observation. A minimum of 3, and ideally 5, video images of the sublingual microcirculation will be taken at each experimental time point, each recorded clip consisting of 100 video frames at a rate of 20 frames per second. Images will be analysed offline using Automated Vascular Analysis software (AVA) V.3.02 (Microvision Medical, The Netherlands). The following data will be obtained: total vessel density (TTD), perfused vessel density (PVD), microvascular flow index (MFI) and microvascular heterogeneity index (MHI).

#### Renal artery Doppler: Haemodynamic assessment of the renal microcirculation

Renal artery ultrasound will be performed using an Affiniti Ultrasound System (Philips, UK) and contrast enhancement. Firstly, the diameter of the renal artery will be measured. Subsequently, pulsed wave Doppler will be used to quantify time-averaged velocity (TAV) within the artery at the same location as the diameter measurement. TAV will then be used to calculate renal blood flow. In addition, intrarenal arterial assessment will be performed using pulsed-wave Doppler at the corticomedullary junction. The peak systolic and end diastolic arterial velocities will be measured during the cardiac cycle and renal arterial resistive index (RI = (peak systolic velocity–end diastolic velocity) / peak systolic velocity) calculated.

#### Transthoracic echocardiography: Haemodynamic assessment of the systemic microcirculation

Transthoracic echocardiography (TTE) will be performed using an Affiniti Ultrasound System (Philips, UK) and standard windows obtained (parasternal long axis, parasternal short axis, Apical 4 chamber, Apical 5 chamber, Apical 2 chamber, subcostal 4 chamber). The TTE, along with vital signs and standard intensive care patient monitoring, will be used to quantify macrocirculatory changes and cardiac output.

#### Urinary oxygen tension

Assessment of urinary oxygen tension will be performed using the OxyLite^™^ single channel dissolved oxygen (pO2) and temperature monitor (Oxford Optronix, UK). Following allocation, all participants will have a sterile oxygen sensor inserted into the lumen of their standard Foley urinary catheter such that the sensor sits at the catheter tip. Urinary pO2 will be recorded for the duration of the study alongside hourly urine output and, at completion, the sensor will be removed.

#### Venous excess ultrasound

Assessment of the inferior vena cava (IVC), portal, hepatic and renal veins will be performed using an Affiniti Ultrasound System (Philips, UK) and a phased-array or curvilinear transducer. Firstly, the IVC will be measured in its intrahepatic portion in either longitudinal or transverse orientation. Hepatic venous flow will then be imaged as the vein drains into the IVC. Colour Doppler will be applied to help identify sonoanatomy and aid optimal placement of a pulsed-wave Doppler gate. This will then be utilised to assess the venous Doppler waveform. Next, portal vein Doppler assessment will be performed. The portal vein is identified by its position with confirmation using pulsed-wave Doppler to differentiate portal venous flow signature (monophasic to biphasic) from the pattern seen in the hepatic artery (sharp systolic upstroke) and the hepatic veins (triphasic). Blood flow velocity in the portal vein usually ranges from 10 to 30 cm/s, so Doppler scale will be adjusted to obtain the best velocity differentiation with minimal noise (usually in the 20–40 cm/s or 0.2–0.4 m/s range). The peak (VMax) and the minimum (VMin) velocities during the cardiac cycle will be recorded. The pulsatility fraction (PF) will be subsequently calculated as follows: PF (%) = 100 (VMax—Vmin / Vmax). Finally, intrarenal venous assessment will be performed using pulsed-wave Doppler at the corticomedullary junction. Here the venous Doppler waveform will be assessed. From these measures a venous excess ultrasound (VExUS) score will be calculated [22].

#### Blood and urine samples

Blood and urine samples will be collected at the beginning and end of the study period from existing intra-vascular lines and urinary catheters. These samples will be processed, stored and used for biomarker analysis. Biomarkers tested will include tissue inhibitor of metalloproteinases-2 (TIMP-2) and insulin-like growth factor binding protein-7 (IGFBP-7): regulatory proteins involved in initiating cell cycle arrest and associated with AKI. In addition, biomarkers of vascular changes and inflammation will be measured, including angiopoietin 1 & 2, syndecan 1, interlukin (IL) 6 & 8 and soluble tumour necrosis factor receptor (sTNFR).

### Outcome measures

#### Primary outcome measure

- Absolute cortical mean transit time (mTT) at the T_4_ (+24 hours) timepoint.

#### Secondary outcome measures

- CEUS derived mTT, perfusion index (PI) and wash in rate (WiR) at the T_3_ (+1 hour) and T_4_ (+24 hours) timepoints.
- IDF video microscopy derived perfused vessel density (PVD) and microvascular flow index (MFI) between treatment groups at the T_3_ (+1 hour) and T_4_ (+24 hours) timepoints.
- TTE derived measures of cardiac output and ventricular function between treatment groups at the T_3_ (+1 hour) and T_4_ (+24 hours) timepoints.
- Mean urinary pO2 across 24 hours study period.
- Tissue inhibitor of metalloproteinases-2 (TIMP-2) and insulin-like growth factor binding protein-7 (IGFBP-7) at 24 hours timepoint.

#### Exploratory outcome measures

- Biomarkers of inflammation and endothelial activation/function (syndecan 1, angiopoietin 1:2, IL-6, IL-8 and sTNFR).

### Data management

The study complies with the principles of the Data Protection Act, 2018. Study investigators will at all times act to preserve confidentiality of patient identifiable information including through de-identification of all retained data.

Upon study enrolment, all participants will be assigned a study number. Deidentified data for each participant will be entered onto a paper case report form (CRF) before transfer to a secure electronic spreadsheet. All physical data, such as the CRFs and consent forms, will be securely stored in a locked research office. All electronic data will be maintained on a secure electronic database accessible only by members of the research team.

### Safety considerations

There are two identified safety considerations associated with this interventional study; the use of the intravenous contrast agent and the use of vasoactive drugs.

The intravenous contrast agent Sonovue^™^ consists of a solution of stabilised microbubbles filled with sulphur hexaflouride. The safety profile of this agent is good and has been extensively reported. Post marketing surveillance provided by the manufacturer reveals use in 2,447,083 patients between 2001 and 2012. Three hundred and twenty two (0.016%) serious adverse reactions were reported, although causality was not clearly established in every case. There have been no fatal reactions reported. Two large retrospective studies on the safety of Sonovue^™^ have yielded either similar or lower serious adverse event (SAE) rates. A recent study of 49,100 patients undergoing Sonovue^™^ enhanced imaging reported 43 (0.088%) adverse events (AEs), of which 7 (0.014%) were serious [23]. In another series of 23,188 administrations there were a total of 29 (0.12%) reported AEs but only 2 (0.009%) of these were classified as serious [24]. SAEs are described as involving bronchospasm, hypotension, rash, drowsiness, convulsions or an anaphylactic reaction. These events were all either short lived or rapidly and completely resolved with treatment. The non-serious AEs are reported to include dizziness, paraesthesia, pruritis, headache, abdominal discomfort and rash.

The safety of both interventional vasoactive agents has been previously tested. Vasopressin is already in widespread clinical use and endorsed by international guidelines [16]. A meta-analysis of the benefit and harm profile of vasopressin compared to other vasoactive agents in septic shock demonstrated safety in terms of mortality, SAEs, mesenteric ischaemia, arrhythmias and acute coronary syndrome. However, vasopressin use was associated with a higher incidence of digital ischaemia [25]. Angiotensin II has been assessed for safety in the ATHOS 3 trial. The results of this study showed no difference in the rates of tachydysrhythmias, peripheral, mesenteric or myocardial ischaemia in critically ill patients with vasoplegic shock treated with Angiotensin II versus placebo. Although there was a slightly higher rate of deep vein thrombosis (DVT) in the angiotensin II group, all patients in this study will be receiving appropriate DVT prophylaxis [17].

Adverse events and SAEs will be reported in the participant’s medical notes and on the study report form. In addition, the principal investigator (PI) will be informed within 24 hours. The PI will assess the event for relatedness and expectedness. In cases where the PI decides that the event was related to contrast administration, interventional vasoactive drugs or is unable to positively exclude such associations, the participant will be withdrawn from the study. Any SAEs related to the administration of contrast or interventional vasoactive agent will be reported to the sponsor and manufacturer within one working day and the REC in an expedited fashion within 15 days.

### Statistical analysis plan

Continuous data will be examined for normality. Reporting will be as mean ± 1 standard deviation (SD) or median and interquartile range (IQR) for normal and non-normal data, respectively. Nominal data will be reported as number and percentage. Longitudinal data will be compared between groups using appropriate statistical tests, for example analysis of variance, with post hoc testing using Tukey’s or Kruskal-Wallis tests depending on data distribution. Relationships between variables will also be assessed using appropriate tests, for example linear regression analysis. Statistical support will be provided by our institution as necessary.

### Ethical considerations and declarations

This study has been approved by the London–South East REC (23/LO/0868). Ethics Committee approved protocol is at S3 File. Due to the nature of septic shock and the fact that patients are, by definition, critically unwell, an emergency waiver of consent has been approved by the REC. After capacity is regained, consent will be sought from patients in accordance with the Mental Capacity Act (2019). All ethical procedures followed will be based on the guidance provided in the Mental Capacity Act 2019 and adhere to the principals laid down within the Declaration of Helsinki. The REPERFUSE study will be conducted in accordance with the approved trial protocol, Good Clinical Practice guidelines, the Data Protection Act (1998) and the Mental Capacity Act (2005). The principal investigator will submit annual progress reports and all protocol amendments to the REC for review.

The authors declare no individual competing interests. The study sponsor and funder had no role in the design of this study. Neither sponsor nor funder will have a role in the study execution, analyses, data interpretation or decision to submit results. A condition of European Society of Intensive Care Medicine (ESICM) funding, the main analysis of this study will be submitted to the Intensive Care Medicine journal for consideration of publication in the first instance.

### Status and timeline of study

Study enrolment is due to commence in the first quarter of 2024 and run until the 45 participant sample size is reached.

## Discussion

Vasopressin is thought to be relatively deficient in septic shock [26]. Systemically, vasopressin acts via vasopressin 1 receptors (V1) in vascular smooth muscle with resultant vasoconstriction. In the kidneys, activity on the vasopressin 2 receptors (V2) contributes to body water homeostasis. However, *in vitro* studies suggest vasopressin also has an effect on intrarenal haemodynamics with efferent arteriole constriction [27]. This might explain the results of septic animal studies where vasopressin was seen to improve urine output and creatinine clearance not explained by changes in renal blood flow [28]. Subsequent large clinical studies of vasopressin have, however, failed to demonstrate either mortality benefit or reduction in renal failure, despite an association with reduced requirements for renal replacement therapy (RRT) [25, 29, 30].

Angiotensin II acts as a vasoconstrictor both systemically, but also at the glomerular level where it is thought to cause a greater increase in efferent than afferent arteriole resistance [31]. Similar to vasopressin, septic animal studies of angiotensin II have demonstrated an improvement in both urine output and creatinine clearance with its administration. Again, these results were not explained purely by changes in MAP or renal macrovascular flow [8]. Although not powered to detect mortality effects, angiotensin II has been studied in a large clinical trial of human vasodilatory shock [17]. Post-hoc analysis of the subpopulation from this study with sustained AKI requiring RRT demonstrated patients randomised to angiotensin II had faster renal recovery and better survival compared to the placebo group [32].

Despite the association between the use of vasopressin and angiotensin II in septic shock and favourable renal outcomes, mechanistic understanding of the effects of these vasopressor on intra-renal perfusion remains incomplete. This study, through the use of the emerging imaging technique of CEUS, aims to contribute to this understanding.

This study also aims to assess the utility of urinary pO2 monitoring in patients with septic shock. In septic animal studies, medullary hypoxia measured via urinary pO2 has been shown to be associated with the development of AKI [33]. These changes in urinary oxygen tension are observed before changes in other measures of renal functional impairment [34]. However, clinical studies of urinary pO2 in septic shock are limited despite the prevalence of AKI in this population. There is also concern that the oligo-anuria seen in these patients may limit the utility of this technique due to the increased error that can result from low urinary flow rates [18]. In this study, post hoc analysis of urinary pO2 alongside hourly urine output will be undertaken to allow assessment of the flow rate related inaccuracy of this measure. If urinary oxygen tension is to become a useful monitoring modality in the critically ill, further studies such as this are required.

### Limitations

Limitations of this mechanistically focussed randomised control trial include the potential for the introduction of performance, detection or attrition biases. In this study, the participants and attending healthcare professionals are not blinded to vasopressor allocation. Due to their clinical condition and physiological measures, it is not anticipated that unblinded participants will cause any issue. For healthcare professionals and investigators this, alongside detection bias, is mitigated as far as possible by the use of objective outcomes and blinding of offline outcome assessment for all microcirculatory measures. When studying the critically ill there is always a small risk of attrition bias from participant demise or withheld deferred consent. This is accounted for as far as possible through the short study window and exclusion of patients with an expected lifespan less than 24 hours.

### Patient and public involvement

The research question developed from previous work within our group and the published research priorities of relevant international organisations. This, and other proposed studies, were presented to the King’s College Hospital Critical Care Patients and Relatives Advisory Group. This group found the risk profile to patients to be acceptable. In addition, they found the study important and interesting, particularly the use of ultrasound imaging. We have made changes to our protocol and patient facing documentation based on the feedback we received from this group. Due to the critically ill nature of the patients being enrolled in this study, we have not involved patients in the study recruitment, but the conduct has been assessed and approved by the REC. The results of this study will be published through open access but not directly distributed to participants.

### Dissemination plans

The results of the REPERFUSE study will be widely and actively disseminated through publication in peer reviewed medical journals and presentations at national and international meetings.

### Amendment plans

All amendments to the study protocol will be tracked, dated and submitted for review and approval where required. Any deviation from the protocol in the study analysis will be explicitly reported and justified.

## Supporting information

S1 File Trial registration data. (DOCX)

S2 File SPIRIT checklist. (DOCX)

S3 File REC approved study protocol. (DOCX)
