# Supplementary material for: The REPERFUSE study protocol: The effects of vasopressor therapy on renal perfusion in patients with septic shock—A mechanistically focused randomised control trial
Source: PLoS One. 2024 Jun 13;19(6):e0304227. doi: 10.1371/journal.pone.0304227 (PMC11175393; doi:10.1371/journal.pone.0304227)
Supplement: S1 File — (DOCX) [file pone.0304227.s001.docx]

**Trial registration data**

| Trial registration | Clinical Trials.gov NCT06234592, 30th Jan 24 |
| --- | --- |
| Funding | The Medical Directorate of the Defence Medical Services, part of the UK Ministry of Defence, and the European Society of Intensive Care Medicine |
| Sponsor | King’s College Hospital NHS Foundation Trust |
| Contact for public and scientific queries | RMcD (rory.mcdonald@nhs.net) |
| Public and scientific title | The effect of vasopressor therapy on renal perfusion in patients with septic shock – a mechanistically focussed randomized control study. (short title: REPERFUSE) |
| Country of recruitment | United Kingdom |
| Health conditions studied | Adult patients with septic shock |
| Interventions | Vasopressin and angiotensin II  Noradrenaline (standard care) |
| Main inclusion/exclusion criteria | Inclusion:  Age >18 years  Within 48hrs of ICU admission  Evidence of suspected or confirmed infection  Sequential Organ Failure Assessment (SOFA) score increase of 2 or more (assuming baseline 0 if no previous measures)  Requiring noradrenaline as the sole vasopressor agent in a dose of > 0.1mcg/kg/min  Lactate >2mmol/l at any stage prior to randomisation  Exclusion:  Known intolerance to Sonovue™ contrast medium, Vasopressin or Angiotensin II  Patients receiving other vasoactive drugs in addition to noradrenaline  Patients with CKD stage 4 or 5  Patients receiving ECMO  Patients with acute occlusive coronary syndromes requiring intervention  Patients with mesenteric ischaemia  Patients with history or presence of aortic dissection or abdominal aortic aneurysm  Patients with Raynaud’s syndrome or acute vaso-occlusive conditions  Pregnancy  Patients with an expected life span of <24hrs in whom the primary treatment intent is palliative |
| Study type | Randomised control trial |
| Date of first enrolment | First quarter 2024 |
| Target sample size | 45 |
| Recruitment status | Pre-recruitment |
| Primary outcome | Contrast enhanced ultrasound (CEUS) derived cortical mean transit time (mTT) at 24hr timepoint |
| Key secondary outcomes | mTT, cortical perfusion index (PI) and cortical wash in rate (WiR) at T+1hr and T+24hrs.  Mean urinary pO2 across 24hr study period.  Tissue inhibitor of metalloproteinases 2 (TIMP-2) and Insulin-like growth factor-binding protein 7 (IGFBP-7) at 24hr timepoint. |
