# Supplementary material for: The REPERFUSE study protocol: The effects of vasopressor therapy on renal perfusion in patients with septic shock—A mechanistically focused randomised control trial
Source: PLoS One. 2024 Jun 13;19(6):e0304227. doi: 10.1371/journal.pone.0304227 (PMC11175393; doi:10.1371/journal.pone.0304227)
Supplement: S3 File — (DOCX) [file pone.0304227.s003.docx]

**The effect of vasopressor therapy on renal perfusion in patients with septic shock – a mechanistically focussed randomized control study**

**Short Title: REPERFUSE**

**Protocol Version 1.1 dated 16 October 2023**

**REC reference: 23/LO/0868**

**IRAS reference: 328797**

**NIHR Portfolio CPMS ID: XXXX**

**Sponsor: King's College Hospital NHS Foundation Trust**

**Funders: Royal Centre for Defence Medicine, European Society of Intensive Care Medicine**

**CI: Prof. Sam Hutchings**

**Contents**

| **STUDY OVERVIEW** | |
| --- | --- |
| Full title | The effect of vasopressor therapy on renal perfusion in patients with septic shock – a mechanistically focussed randomized control study. Short title - REPERFUSE |
| Objectives | To investigate the effect of changing blood pressure supportive therapy on blood flow to the kidney. |
| Type of trial | Randomised control study |
| Trial design and methods | Patients will be randomised to 3 groups: 1. Noradrenaline alone (standard care) 2. Noradrenaline + Vasopressin, 3. Noradrenaline + Angiotensin II. Drugs will be targeted to a clinically determined MAP with the aim of maximising drug dose and minimising noradrenaline. Patients will receive the intervention for 24 h. Serial measurements of renal cortical blood flow will be made using contrast ultrasound alongside measures of other haemodynamic variables |
| Health condition(s) or problem(s) studied | Adult patients with septic shock |
| Target sample size | 45 |
| Trial duration per participant: | 24 hours |
| Main inclusion/exclusion criteria: | Inclusion:  Age > 18 years  Within 48 hours of ICU admission  Evidence of suspected or confirmed infection  Serial Organ Failure Assessment (SOFA) score increase of 2 or more (assuming baseline 0 if no previous measures)  Requiring noradrenaline as the sole vasopressor agent in a dose of > 0.1 mcg/kg/min  Lactate > 2 mmol/l at any stage prior to randomisation  Exclusion:  Known intolerance to Sonovue™ contrast medium, Vasopressin or Angiotensin II  Patients receiving other vasoactive drugs in addition to noradrenaline  Patients with known end stage renal failure or CKD stage 4  Patients receiving ECMO  Patients with acute occlusive coronary syndromes requiring intervention  Patients with mesenteric ischaemia  Patients with history of or presence of aortic dissection or abdominal aortic aneurysm  Patients with Raynauds syndrome or acute vaso occlusive conditions  Pregnancy  Patients with an expected life span of < 24 h in whom the primary treatment intent is palliative |
| Statistical methodology and analysis: | Longitudinal data will be compared between groups and over time using analysis of variance with post hoc testing using Tukey’s or Kruskal- Wallis tests depending on distribution of the data . Relationships between variables will be assessed using linear regression analysis. |
| **STUDY TIMELINES** | |
| Study Duration/length | 24 months |
| Expected Start Date | Q1 2024 |
| End of Study definition and anticipated date | Last patient data analysed. June 2026 |
| Key Study milestones | Funding obtained March 2023  Approvals complete Q3 2023  First patient recruited Jan 2024  Final patient recruited Jan 2026  Data analysis complete Jun 2026 |
| **STORAGE of SAMPLES**  **(if applicable)** | |
| Human tissue samples | Blood and urine |
| Data collected / Storage | Stored frozen. Utilised for ELISA analysis as part of current study only then destroyed |
| **Device Information** | |
| Device Name | Oxylite |
| Manufacturer Name | Oxford Optronics |
| Principle Intended Use | Assessment of urinary pO2 |
| Length of time the device has been in use |  |

[1. Background and rationale 6](#_Toc148343254)

[2. Aims 7](#_Toc148343255)

[3 Study design 7](#_Toc148343256)

[3.1 Overview 7](#_Toc148343257)

[3.2 Setting 7](#_Toc148343259)

[Tertiary intensive care. 7](#_Toc148343260)

[3.3 Population 7](#_Toc148343261)

[Adult patients with septic shock. 7](#_Toc148343262)

[3.3.1 Inclusion Criteria 7](#_Toc148343263)

[3.3.2 Exclusion criteria 7](#_Toc148343264)

[3.4 Co-enrolment 8](#_Toc148343265)

[3.5 Screening 8](#_Toc148343266)

[3.6 Treatment assignment 8](#_Toc148343267)

[3.7 Recruitment and consent 8](#_Toc148343268)

[3.7.1 Overview & rationale 8](#_Toc148343269)

[3.7.2 Informed deferred consent 9](#_Toc148343270)

[3.7.3 Role of Personal Consultee 10](#_Toc148343271)

[3.7.4 Event of patient death 10](#_Toc148343272)

[3.7.5 Event of discharge / transfer prior to recovery of capacity 11](#_Toc148343273)

[3.8 Study drug administration 11](#_Toc148343274)

[3.8.1 Initiation of study vasopressors 11](#_Toc148343275)

[3.8.2 Discontinuation of vasopressor drugs 12](#_Toc148343276)

[3.8.3 Addition of other vasopresssors 12](#_Toc148343277)

[3.8.4 Selection of MAP target 12](#_Toc148343278)

[3.9 Data collection 12](#_Toc148343279)

[3.9.1 Trans thoracic echocardiography 13](#_Toc148343280)

[3.9.2 Incident dark field video-microscopy 13](#_Toc148343281)

[3.9.3 Renal ultrasound including use of contrast 13](#_Toc148343282)

[3.9.4 Abdominal Ultrasound 14](#_Toc148343283)

[3.10 Outcome measures 15](#_Toc148343284)

[3.11 Data management 15](#_Toc148343285)

[3.12 Device Information 16](#_Toc148343286)

[4 Safety Monitoring & Reporting 16](#_Toc148343287)

[4.1 Safety issues relating to Sonovue contrast agent 16](#_Toc148343288)

[4.1 Safety Issues relating to vasoactive drugs 17](#_Toc148343289)

[4.2 Definitions 17](#_Toc148343290)

[5. Study closure 19](#_Toc148343303)

[5.1 Data archiving 19](#_Toc148343304)

[6. Statistics and data analysis 20](#_Toc148343305)

[6.1 Analysis of microcirculation images 20](#_Toc148343306)

[6.2 Analysis of contrast ultrasound images 20](#_Toc148343307)

[6.3 Statistical analysis 20](#_Toc148343308)

[7. Ethical compliance and standards 21](#_Toc148343309)

[7.1 Study registration 21](#_Toc148343310)

[7.2 Central ethical compliance 21](#_Toc148343311)

[8. Data handling and management 21](#_Toc148343312)

[9. Storage of Biological Samples 22](#_Toc148343313)

[10. Sponsorship and Funding 22](#_Toc148343314)

[10.1 Sponsoring Organisation 22](#_Toc148343315)

[10.2 Funder 22](#_Toc148343316)

[11. Dissemination of results 22](#_Toc148343317)

[12. Peer and regulatory review 22](#_Toc148343318)

[13. Indemnity Arrangements 23](#_Toc148343319)

[14. References 23](#_Toc148343320)

# 1. Background and rationale

Critically ill patients with septic shock are at a greatly increased risk of developing acute renal failure (ARF), the incidence of which may be as high as 51%^1^ Historically AKI in the setting of shocked critically ill patients has been considered predominantly a result of systemic factors which lead to a reduction in global and regional blood flow to the kidneys as well as part of the generalised endotheliopathy seen in shock states^2^. However, recent experimental and clinical studies have provided evidence that global systemic and renal blood flow is often preserved or even increased, at least after initial resuscitation has concluded ^3,4^. New techniques, particularly contrast enhanced ultrasound (CEUS) have provided an ability to study renal cortical small vessel perfusion in real time at the bedside and have demonstrated that perfusion is often profoundly impaired, even in the presence of normal renal arterial blood flow and venous tone. In addition, recent work from our research group has suggested that this impaired small vessel flow was not associated with other markers of systemic inflammation or wider microcirculatory impairment, raising the possibility of an intrinsic renal mechanism involving shunting or preferential afferent vs efferent arteriolar glomerular vasoconstriction^4^. This later hypothesis has been supported by several large studies which have suggested that shocked patients treated with the addition of vasopressor agents, such as angiotensin II and vasopressin, which potentially cause relatively more efferent than afferent arteriolar vasoconstriction, have improved renal outcomes^5,6^. However, the evidence from these studies is not conclusive and lacks a firm mechanistic basis.

CEUS is ultimately a research tool, allowing the testing of mechanistic hypotheses in small numbers of patients, but ultimately too time consuming and user dependent to be translated into mainstream clinical practice. There is currently no monitor with the proven ability to detect changes in renal perfusion in real time in critically ill patients. The ability to measure oxygen tension in urine, which has been utilised in both experimental and clinical studies, may provide a solution in clinical practice^7^. Oximeters that can be easily inserted through standard urethral catheters are commercially available. Should the output of such a monitor be correlated with renal perfusion measured using CEUS then the possibility of bringing such devices into routine clinical practice becomes possible. Such a device could potentially provide both an early warning signal of AKI and allow for the assessment of therapy

#

# 2. Aims

1. To assess the effect of differential vasopressor therapies (noradrenaline, vasopressin and angiotensin II) on renal cortical perfusion in patients with septic shock
2. To assess the relationship between continuous urinary oximetry and renal cortical perfusion

# 3 Study design

## 3.1 Overview

Randomized control study. Patients will be randomized to receive either noradrenaline alone or noradrenaline and additional vasopressor (vasopressin / angiotensin II) for 24 hours. Assessment of renal cortical perfusion will be conducted using CEUS at 3 time points. Systemic haemodynamic and sublingual microcirculatory parameters will be collected to allow a mechanistic understanding of the observed changes in renal cortical perfusion.

## 3.2 Setting

Tertiary intensive care.

## 3.3 Population

Adult patients with septic shock.

### 3.3.1 Inclusion Criteria

Age > 18 years

Within 48 hours of ICU admission

Evidence of suspected or confirmed infection

Serial Organ Failure Assessment (SOFA) score increase of 2 or more (assuming baseline 0 if no previous measures)

Requiring noradrenaline as the sole vasopressor agent in a dose of > 0.1 mcg/kg/min

Lactate > 2 mmol/l at any stage prior to randomisation

### 3.3.2 Exclusion criteria

Known intolerance to Sonovue™ contrast medium, Vasopressin or Angiotensin II

Patients receiving other vasoactive drugs in addition to noradrenaline

Patients with known end stage renal failure or CKD stage 4

Patients receiving ECMO

Patients with acute occlusive coronary syndromes requiring intervention

Patients with mesenteric ischaemia

Patients with history of or presence of aortic dissection or abdominal aortic aneurysm

Patients with Raynauds syndrome or acute vaso occlusive conditions

Pregnancy

Patients with an expected life span of < 24 h in whom the primary treatment intent is palliative

## 3.4 Co-enrolment

Co-enrolment is permitted for observational or interventional studies with the agreement of both PIs.

## 3.5 Screening

Screening will be conducted by study investigators using remote access to the ICU clinical information system. All study investigators are intensive care clinicians at the relevant institution and have routine access to patients clinical records as part of their clinical role. Following identification of patients who meet the inclusion and exclusion criteria the patients attending consultant will be approached to provide an opinion.

## 3.6 Treatment assignment

Enrolled patients will be randomly allocated to one of three groups using a random permuted block allocation with a block size of 6. Sealed Envelope Simple + software will be used to allocate patients to groups. Groups will be:

- Noradrenaline alone (standard care)
- Noradrenaline + vasopressin
- Noradrenaline + angiotensin II

## 3.7 Recruitment and consent

### 3.7.1 Overview & rationale

Patients with severe sepsis, eligible for inclusion in the study, are critically unwell, invariably receiving sedative medications and frequently exhibiting both a reduced level of consciousness and inability to effectively communicate. For all these reasons, they typically lack capacity to provide prior informed consent. The point of maximal scientific interest and potential for future treatment developments occurs in the first hours of critical illness during this period of mental incapacity. For these reasons any attempt to obtain either prior informed consent from the patient, or the opinion of their Personal Consultee (i.e. relative or close friend), prior to study enrolment would be inappropriate.

In view of these considerations, once an eligible patient is identified for the trial (i.e. the patient meets the inclusion criteria and does not meet any exclusion criteria), a registered medical practitioner independent of the study will be asked to give permission for enrolment. This medical practitioner will usually be the patients attending consultant. If permission is granted they will be enrolled into the study. This method is known as ‘deferred consent’ or ‘research without prior consent’ and is recognised in law. This process will be covered by an emergency waiver of consent under the Mental Capacity Act approved by London South East Research Ethics Committee.

### 3.7.2 Informed deferred consent

Following enrolment, patients will be approached once they have been deemed to have full capacity to provide informed deferred consent. A Participant Information Sheet (PIS) will be provided to the patient. The PIS will provide information about the purpose of the study, what participation means for the patient, confidentiality and data security, and the future availability of the trial results.

A Consent Form will be provided indicating that: the information given, orally and in writing, has been read and understood; participation is voluntary and can be withdrawn at any time without consequence; and that consent is given for access to medical records for data collection. The Consent Form will also cover ongoing data collection and follow-up.

Patients will be given time to read the PIS and have an opportunity to ask any questions they may have about participation in the study. After verifying that the PIS and Consent Form are understood, the person seeking consent will invite the patient to sign the Consent Form and will then add their own name and countersign it. A copy will be given to the patient, a copy placed in the patient’s medical notes and the original kept in the Investigator Site File. If the patient is unable to physically sign the Consent Form (e.g. due to physical incapacity), an independent witness can sign on their behalf.

### 3.7.3 Role of Personal Consultee

As outlined in Section 3.7.1 it will not be possible to involve patients in the consenting process at study recruitment. Instead, consent will be obtained from patients once they have stabilised and are deemed to have capacity. Furthermore, given that the study intervention is confined to 24 hour after recruitment it will not often be possible or appropriate to consult with a personal consultee (a friend / relative of the patient) before the intervention is underway.

However, within a reasonable time frame after study enrolment, a delegated member of the research team will approach a close friend or relative, known as a personal consultee to seek advice as to the patients’ likely wishes and feelings regarding participating in research. Ideally, this approach would take place once the patient’s medical situation is no longer an emergency and initial meetings between the consultee and treating clinicians have occurred.

The Personal Consultee will be provided with a Personal Consultee Information Sheet, containing all of the information provided on the PIS, supplemented by information about why the Personal Consultee has been approached at this stage. A Personal Consultee Opinion Form will be provided indicating that: the information given, orally and in writing, has been read and understood; the patients’ participation is voluntary and can be withdrawn at any time without consequence; and that, in the Personal Consultees opinion, the patient would not object to taking part in research. Personal Consultees will also be asked to indicate on the Personal Consultee Opinion Form whether, in their opinion, the patient would agree to access to medical records for data collection.

Personal Consultees will be given time to read the Personal Consultee Information Sheet and have an opportunity to ask any questions they may have about the patients’ participation in the study. After verifying that the Personal Consultee Information Sheet and Opinion Form are understood, the person seeking opinion will invite the Personal Consultee to sign the Personal Consultee Opinion Form and will then add their own name and countersign it. A copy will be given to the Personal Consultee, a copy placed in the patient’s medical notes and the original kept in the Site File.

If a Personal Consultee advises that, in their opinion, the patient would not choose to participate in research, then the study intervention will cease including transitioning the patient from the study drugs if these have been administered and no further data collection will occur.

3.7.4 Role of Nominated Consultee

A Nominated Consultee will be approached in the rare situations where no Personal Consultee is available (or one is available, but unwilling to provide an opinion). The Nominated Consultee will be an independent clinician (i.e. not associated with the conduct of the study). Opinion of the Nominated Consultee will be sought in the same manner as for the Personal Consultee.

Upon patient recovery, the patient will be approached directly for informed deferred consent (see section 3.7.2). The patient’s decision will be final, and will supersede the Nominated Consultee, where there is disagreement.

### 3.7.4 Event of patient death

In the situation where the patient dies any data collected will be used in analysis. There will be no requirement to make further approaches to a consultee in this instance.

### 3.7.5 Event of discharge / transfer prior to recovery of capacity

In the rare situation where the patient is transferred to another hospital prior to consent being sought, then the most appropriate member of the site research team will liaise with the receiving hospital to establish at which point capacity has been regained and when discharge is probable. If possible, patients will be approached by researchers in person at the other site to provide informed consent for data use. Patients will not be contacted remotely (e.g. by letter).

## 3.8 Study drug administration

The overall aim is to maximise the dose of the drug whilst reducing the dose of noradrenaline and maintaining the target MAP set by the attending clinician.

Prior to any study interventions patients should have been on a stable dose of noradrenaline for at least one hour.

### 3.8.1 Initiation of study vasopressors

Following collection of baseline measurements patients randomised to receive either vasopressin or angiotensin II will have these infusions commenced using the following dosing regimen:

- Vasopressin: 20 units in 50ml 5% glucose (0.4 IU/ml). Infusion commenced at 0.01 IU/min (1.5ml/hr) and increased every 10 minutes to a dose of 0.04 IU/min (4.5 ml/hr).
- Angiotensin II: 2.5mg in 250mls 0.9% sodium chloride (10,000 ng/ml). Infusion commenced at 20 ng/kg/min and increased by 5ng/kg/min every 5 minutes to reach a target dose of 40 ng/kg/min

All dose calculations will be based on actual body weight.

As the vasopressor drugs are commenced, noradrenaline dosing will be titrated to maintain the target MAP. If the target MAP is achievable with the vasopressor alone then noradrenaline should be discontinued. If the MAP target is exceeded after noradrenaline is discontinued then the study vasopressor should be weaned in accordance with the protocol at 3.8.2.

### 3.8.2 Discontinuation of vasopressor drugs

After completion of the 24 hour study intervention window, or if infusion of the study vasopressor is causing the MAP target to be exceeded then the study vasopressor will be reduced / withdrawn as follows:

- Vasopressin. Reduce dose by 0.01 IU / min every 30 mins until discontinued.
- Angiotensin II. Reduce dose by 5 ng/kg/min every 5 mins until discontinued.

Concurrently noradrenaline will be increased as required to maintain the target MAP.

### 3.8.3 Addition of other vasopresssors

If the attending clinician decides to add vasopressor therapy, for example adding terlipresin to a patient in the noradrenaline only group, during the course of the intervention period then no further study measurements will be made and the deviation from the protocol will be recorded. Subsequent titration / reduction of drugs will be made according to instruction from the attending clinician in this circumstance.

### 3.8.4 Selection of MAP target

Selection of target MAP will be at the discretion of the attending clinician. The MAP target will be recorded on the CRF. MAP targets should not be changed during the intervention period.

## 3.9 Data collection

Data collection will follow the schema in Fig 1


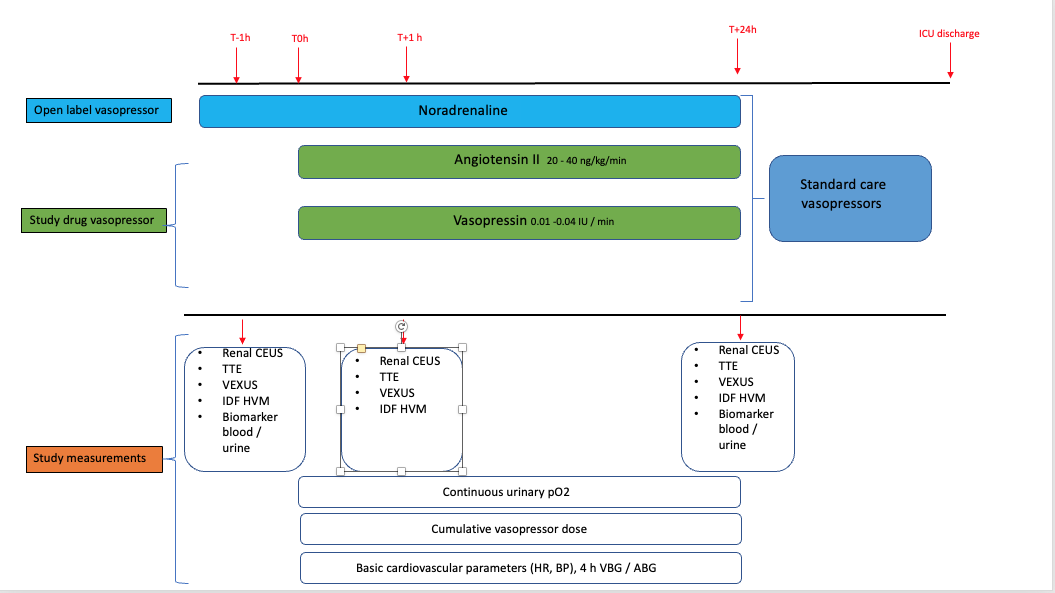


Data collection using the subject devices (cardiac echocardiography, renal contrast enhanced ultrasound, VEXUS ultrasound and sublingual video microscopy) will be undertaken at baseline (T-1h), 1 h after steady state is achieved following institution of study drugs (T +1 h) and 24 h later immediately prior to discontinuing study drugs (T+24h). All procedures will be undertaken by a study investigator specifically trained in the individual techniques.

### 3.9.1 Trans thoracic echocardiography

###

Transthoracic echocardiography will be performed using an Affiniti Ultrasound System (Philips, UK) and standard windows obtained (parasternal long axis, parasternal short axis, Apical 4 chamber, Apical 5 chamber, Apical 2 chamber, subcostal 4 chamber).

### 3.9.2 Incident dark field video-microscopy

Videos of the sublingual microcirculation will be acquired using an Incident Dark Field (IDF) video-microscope (Cytocam, Braedius Medical, Huizen, The Netherlands).

Following suctioning of the oropharynx, a gauze swab will be applied to gently remove saliva from the mucosal surface. The camera probe will be applied to the sublingual area and images selected, taking care to exclude areas of buccal microcirculation with large numbers of looped vessels. The device will be focussed until individual erythrocytes can be visualized within capillaries and the brightness setting adjusted to produce an acceptable degree of contrast between blood vessels and background tissue. At all times pressure artefact will be scrupulously avoided by applying only the minimal amount of pressure necessary to obtain an image. Stopped or reversed flow in larger venules will be taken as a sign of pressure artefact, necessitating adjustment of the camera or selection of a different area of the microcirculation for observation. A minimum of 3, and ideally 5, video images of the sublingual microcirculation will be taken at each experimental time point, each recorded clip consisting of 100 video frames at a rate of 20 frames per second.

Images will be analysed offline using Automated Vascular Analysis software. Images will be identified with the study number and no patient identifiable details will be included.

### 3.9.3 Renal ultrasound including use of contrast

Renal ultrasound, including the use of contrast will be performed using an Affiniti ultrasound system (Philips UK).

Conventional grayscale US imaging will be performed. The investigator will vary the pulse repetition frequency, focal zone, gain, and wall filter as necessary to obtain optimal sonograms in each case. Both kidneys will be visualized and the most accessible chosen to perform the study. Baseline grayscale and colour Doppler sonographic images will be recorded.

A low–mechanical index (MI) technique (range: 0.04 – 0.1) for CEUS will be utilised with MI set at or below 0.10. A continuous infusion of 4.8 mL of SonoVue^TM^ (Bracco SpA, Milan, Italy), contrast agent, will be administered at a rate of 1ml/min.

Images of the entire examination will be digitally recorded. After 2 minutes infusion time steady state will be achieved and 5 high frequency ultrasound pulses will be delivered, one every 30 s. Destruction-replenishment kinetics will be quantified by post-processing, performed offline using VueBoxTM (Bracco Diagnostic Imaging, Switzerland). Cortical regions lying in proximity to the probe with good views and reliably visible reperfusion will be identified as regions of interest and the following variables calculated: cortical mean transit time (CMTT), perfusion index (CPI), wash-in rate (CWIR) and relative blood volume (CRBV).

### 3.9.4 Abdominal Ultrasound

Assessment of the inferior vena cava (IVC), portal, hepatic and splenic vein will be performed.

The IVC will be measured in its intrahepatic portion in either longitudinal or transverse orientation.

The hepatic vein flow will be imaged using a phased-array or curvilinear transducer as it drains into the IVC. The use of colour Doppler to help identify and aid optimal placement of pulsed-wave Doppler for analysis will be utilised.

Portal vein Doppler assessment will be performed using a phased-array or curvilinear transducer positioned in a right posterior-axillary coronal view in the 9th to 11th intercostal spaces. The portal vein is identified by its position with confirmation using pulsed-wave Doppler mode to differentiate portal venous flow signature (monophasic to biphasic) from the pattern seen in the hepatic artery (sharp systolic upstroke) and the hepatic veins (triphasic). Blood flow velocity in the portal vein usually ranges from 10 to 30 cm/s, so Doppler scale will be adjusted to obtain the best velocity differentiation with minimal noise (usually in the 20–40 cm/s or 0.2–0.4 m/s range).

The peak (VMax) and the minimum velocities (VMin) during the cardiac cycle will be recorded. The pulsatility fraction (PF) will be subsequently calculated as follows:

PF% = 100 (VMax -VMin/Vmax)

Intrarenal Doppler assessment will be performed using pulsed wave Doppler waveform at the corticomedullary junction. The peak (VMax) and the minimum arterial velocities (VMin) during the cardiac cycle will be recorded. The renal arterial resistive index (RI=(VMax–VMin)/VMax) will be calculated.

VEXUS scores will be calculated based on the results obtained.

## 3.10 Outcome measures

- Primary:

Cortical mean transit time at 24 h timepoint

- Secondary:

CEUS derived CMTT, CPI,CWIR at T+1h and T+24h

Mean urinary pO2 across 24 h study period

TIMP2/ IGFBP7 at 24 h timepoint

- Exploratory

IDF PVD & MFI between treatment groups at T+1h & T+24h

Biomarkers of inflammation and endothelial activation / function (Syndecan 1, angiopoetin 1:2, IL-6, IL-8)

## 3.11 Data management

All participant data collected will be entered onto a paper CRF before being transferred to an electronic spreadsheet. The PI will oversee and be responsible for data collection, quality and recording.

Security of the electronic spreadsheet is through restricted access permissions. Storage and handling of confidential trial data and documents will be in accordance with the Data Protection Act.

## 3.12 Device Information

The OxyLiteTM Pro 2 is a pO2 and temperature monitor that can be used in conjunction with the NX-LAS-1/O/E probe in order to measure dissolved oxygen concentration. These two devices will be jointly used as part of this study to assess the physiological effects of different vasopressor agents on renal oxygenation in critically ill patients. Within this study, the primary focus of the devices is to provide accurate measurements of urine oxygen concentration.

As the devices are intended for experimental (non-clinical) research applications only, they do not possess approval from a regulatory body for clinical use. It is important to note, however, that they are in conformity with the essential requirements of the Low Voltage Directive 2014/35/EU, Electromagnetic Compatibility Directive 2014/30/EU, and the Restriction of the Use of Certain Hazardous Substances in Electrical and Electronic Equipment Directive 2011/65/EU.

There are no CE marked alternatives available on the market which therefore, highlights the need of these devices for the study.

A risk assessment has been previously conducted to enable the use of these devices within a clinical research environment in the UK (Guy’s and St Thomas’ NHS Foundation Trust).

# 4 Safety Monitoring & Reporting

## 4.1 Safety issues relating to Sonovue contrast agent

Sonovue consists of a solution of stabilised microbubbles filled with sulphur hexaflouride. The safety profile of this agent is good and has been extensively reported. In a series of 23,188 administrations reported in an Italian series there were a total of 29 (0.12%) reported adverse events but only 2 (0.009%) of these were classified as serious. Of the 2 SAEs one manifested as bronchospasm and mild hypotension which recovered within 30 minutes without requiring ventilatory support. The second incident was associated with severe hypotension, rash and drowsiness. Again, this resolved within 30 minutes.

The non-severe AEs reported in this series include dizziness, paraesthesia, itching and rash.

2 smaller case series have reported on the use of Sonovue to assess renal perfusion in critically unwell patients. The first investigated the effect of noradrenaline on renal perfusion (n=12), the second the effect of cardiac surgery on renal perfusion (n=12). No adverse events were reported in these two series.

Post marketing surveillance provided by the manufacturer of Sonovue^TM^ reveals use in 2,447,083 patients between 2001 and 2012. 322 (0.0162%) serious adverse reactions were reported although causality was not clearly established in every case. There have been no fatal reactions reported.

## 4.1 Safety Issues relating to vasoactive drugs

Angiotensin II has been assessed for safety in the ATHOS 3 trial. The results showed no difference in adverse events between critically ill patients with vasoplegic shock treated with Angiotensin II versus placebo. Specifically, there was no difference in the rates of tachydysrhythmias, peripheral ischaemia, intestinal ischaemia or myocardial ischaemia.

Vasopressin was assessed for safety against noradrenaline in four studies. A meta-analysis showed that vasopressin use was associated with a higher incidence of digital ischaemia but a lower incidence of cardiac dysrhythmias. Mesenteric ischaemia and acute coronary syndrome incidents was similar in both treatment groups.

The safety of both of these vasoactive agents has thus been tested in previously studies. It is not the purpose of the current study to acquire further safety data on these drugs which, in the case of vasopressin and its analogues are now in widespread clinical use as part of the treatment of septic shock.

## 4.2 Definitions

**Adverse Event**

An Adverse Event (AE) is any untoward medical occurrence affecting a trial participant during the course of a clinical trial. All eligible study patients are by definition critically unwell with the propensity to develop multiple adverse events which would be difficult to relate to the addition of vasoactive agents or microbubble contrast medium. However, we will actively look for any adverse event which appears temporally related to bubble contrast administration.

Given the safety profile of both angiotensin II and vasopressin and the fact that the later is often part of standard care for patients with septic shock we are not listing any expected adverse events in relation to the addition of either of these agents.

**Serious Adverse Event**

A Serious Adverse Event (SAE) is an adverse event that:

· Results in death

· Is a life-threatening situation

· Requires prolongation of hospitalisation

· Results in persistent or significant disability or incapacity

**Relatedness**

None: there is no evidence of any relationship to the study treatment

Unlikely: there is little evidence to suggest a relationship to the study treatment, and there is another reasonable explanation of the event

Possibly: there is some evidence to suggest a relationship to the study treatment, although the influence of other factors may have contributed to the event

Probably: there is probable evidence to suggest a relationship to the study treatment, and the influence of other factors is unlikely

Definitely: there is clear evidence to suggest a relationship to the study treatment, and other possible contributing factors can be ruled out.

**Expectedness**

Expected:

The following events are defined as expected AEs based on possible manifestations of contrast related allergic reactions:

- Bronchospasm
- Hypotension (fall of > 10 mmHg from pre-administration baseline)
- Rash
- Itching
- Dizziness

Unexpected: Other events not listed above.

## 4.2 Recording and reporting AEs

All SAE/SADE/UADEs need to be reported to the sponsor/legal representative, manufacturer, and R&I within one working day of the research team becoming aware of them.

Reports of related and unexpected SAEs should be submitted to the REC within 15 days of the Chief Investigator becoming aware of the event, using the SAE/SADE report form

All reporting to King's College Hospital NHS Foundation Trust should be by e-mail giving as much information about the incident as possible, and should be signed by the PI or Co-investigator. The SADE reporting form should be used for King's College Hospital NHS Foundation Trust sponsored studies.

The sponsor will undertake an initial review of the information and ensure it is reviewed by the MEMS. Events will be followed up until resolution, any appropriate further information will be sent by the research team in a timely manner.

Reporting to the MHRA will be done in liaison with the Chief Investigator and the Manufacturer.

The Manufacturer has a legal obligation to report all events that need to be reported to the Nominated Competent Authority immediately (without any unjustifiable delay) after a link is established between the event and the device, but no more than:

- 2 days following the awareness of the event for Serious Public Health Threat.
- 10 days following awareness of the event for Death or unanticipated serious deterioration in health.
- 30 days following the awareness of the event for all other event meeting the SAE criteria.

## 4.3 Serious Adverse Events that do not need reporting

Patients recruited to the TARGET UP study are by definition critically unwell and by definition meet the criteria for SAE reporting on a frequent basis. SAEs will only therefore be reported if there is a casual link to the study, in the opinion of the investigator or attending staff, or if the frequency and / or nature of the pattern of SAE is, in the opinion of the investigator or attending clinicians, out of keeping with the expected pattern of illness.

# 5. Study closure

## 5.1 Data archiving

Data will be archived for 3 years after study closure. De-identified data will be held within the Department of Critical Care at King’s College Hospital.

# 6. Statistics and data analysis

All images will be assessed offline by a single operator.

## 6.1 Analysis of microcirculation images

Video microscopy images will be assessed using AVA software to produce the following data:

Total Vessel density (TVD)

Perfused Vessel Density (PVD)

Microvascular Flow Index (MFI)

Microvascular Heterogeneity Index (MHI)

## 6.2 Analysis of contrast ultrasound images

Regions-of-interest (ROIs) will be manually drawn: 1 on an interlobar artery, 1 in the renal cortex, 1 in the renal medulla, and a ROI containing the entire kidney. The ROIs for the cortex and medulla will be identical in size for every clip and drawn at approximately the same depth. For every ROI, the software determined mean pixel intensities proportional to contrast-agent concentration and created a time-intensity curve (TIC).

Based on the time– intensity curve, peak enhancement (PE), wash-in area under the curve (WiAUC), rise time (RT), mean transit time (mTT), time to peak (TTP), wash-in rate (WiR), wash-in perfusion index (WiPI; WiAUC/RT), wash-out area under the curve (WoAUC), total area under the curve (AUC), fall time (FT), and wash-out rate (WoR) were analysed. Parameters related to blood volume are PE, WiAUC, WoAUC and AUC. The other parameters, i.e. RT, mTT, TTP, WiR, WiPI, FT, WoR, are related to blood velocity. Renal perfusion could also be assessed based on the Perfusion Index through CEUS replenish kinetics.

## 6.3 Statistical analysis

Longitudinal data will be compared between groups and over time using analysis of variance with post hoc testing using Tukey’s or Kruskal- Wallis tests depending on distribution of the data . Relationships between variables will be assessed using linear regression analysis.

# 7. Ethical compliance and standards

Patients eligible for enrolment in this study will lack capacity to provide informed consent. The ethical procedures followed by this study will be based on the guidance provided in the Mental Capacity Act 2005 and adhere to the principals laid down within the Declaration of Helsinki.

The REPERFUSE study will be conducted in accordance with the approved trial protocol, ICH-GCP guidelines, the Data Protection Act (1998) and the Mental Capacity Act (2005)

## 7.1 Study registration

The study will be registered with an appropriate clinical trial registry prior to commencement.

## 7.2 Central ethical compliance

The study will be reviewed by a REC through the normal IRAS process

The PI will submit annual progress reports and all protocol amendments to the REC for review.

# 8. Data handling and management

The study complies with the principles of the Data Protection Act, 2018. At all times researchers will act to preserve the confidentiality of patient identifiable data.

Patients will be de-identified by allocation of a unique study number and collected data will be referred to this study number rather than to personal identifiable information. Personal data, including full name, contact details, date of birth and NHS number will be required to successfully follow-up enrolled patients and will be linked to collected data on a separate electronic spreadsheet. Only members of the immediate research team will have access to personal identifiable data. Personal data will not be retained after follow up is complete and will be deleted at this time. The research team will act to preserve participant confidentiality and will not disclose or reproduce any information by which participants could be identified.

All physical data, such as Clinical Report Forms & Consent Forms will be securely stored in a locked research office.

All electronic data will be maintained on a secure electronic database accessible only by members of the research team.

De-identified data will be retained indefinitely within the sponsors institution.

# 9. Storage of Biological Samples

In the study, blood and urine will be collected from patients in accordance with the patient consent form and patient information sheet. Samples will be processed, stored and disposed in accordance with all applicable legal and regulatory requirements, including the Human Tissue Act 2004 and any amendments thereafter.

Samples will be de-identified by allocation of a unique study number.

Samples will be stored within the Clinical Research Facility at King's College Hospital.

De-identified samples may be shared with other academic institutions. Permission for this will be explicitly sought during the initial consent process.

# 10. Sponsorship and Funding

## 10.1 Sponsoring Organisation

The study is sponsored by King's College Hospital NHS Foundation Trust.

## 10.2 Funder

The study is funded by the Medical Directorate of the Defence Medical Services, part of the UK Ministry of Defence and the European Society of Intensive Care Medicine.

# 11. Dissemination of results

The results of the REPERFUSE study will be widely and actively disseminated through publication in peer reviewed medical journals and presentations at national and international meetings.

# 12. Peer and regulatory review

The study has been peer reviewed in accordance with the requirements outlined by the Sponsor.

The Sponsor considers the procedure for obtaining funding from the Research Directorate of the Defence Medical Services and the European Society of Intensive Care Medicine to be of sufficient rigour and independence to be considered an adequate peer review.

The study was considered and received a favourable opinion from the London South East Research Ethics Committee (23/LO/0868).

# 13. Indemnity Arrangements

King's College Hospital will provide NHS indemnity cover for negligent harm, as appropriate and is not in the position to indemnify for non-negligent harm. NHS indemnity arrangements do not extend to non-negligent harm and NHS bodies cannot purchase commercial insurance for this purpose; it cannot give advance undertaking to pay compensation when there is no negligence attributable to their vicarious liability. The Trust will only extend NHS indemnity cover for negligent harm to its employees, both substantive and honorary, conducting research studies that have been approved by the R&D Department. The Trust cannot accept liability for any activity that has not been properly registered and Trust approved. Potential claims should be reported immediately to the R&I Office.

# 14. References

1. Uchino S, Kellum JA, Bellomo R, Doig GS, Morimatsu H, Morgera S, et al. Acute renal failure in critically ill patients: a multinational, multicenter study. JAMA. American Medical Association; 2005 Aug 17;294(7):813–8.
2. Bellomo R, Kellum JA, Ronco C, Wald R, Martensson J, Maiden M, et al. Acute kidney injury in sepsis. Intensive Care Med. Springer Berlin Heidelberg; 2017 Mar 31;43(6):816–28.
3. Calzavacca P, Evans RG, Bailey M, Bellomo R, May CN. Cortical and Medullary Tissue Perfusion and Oxygenation in Experimental Septic Acute Kidney Injury. Critical Care Medicine. 2015 Oct;43(10):e431–9.
4. Watchorn, J., Huang, D., Bramham, K. & Hutchings, S. Decreased renal cortical perfusion, independent of changes in renal blood flow and sublingual microcirculatory impairment, is associated with the severity of acute kidney injury in patients with septic shock. *Crit Care* **26**, 261 (2022).
5. Tumlin JA, Murugan R, Deane AM, Ostermann M, Busse LW, Ham KR, Kashani K, Szerlip HM, Prowle JR, Bihorac A, Finkel KW, Zarbock A, Forni LG, Lynch SJ, Jensen J, Kroll S, Chawla LS, Tidmarsh GF, Bellomo R; Angiotensin II for the Treatment of High-Output Shock 3 (ATHOS-3) Investigators. Outcomes in Patients with Vasodilatory Shock and Renal Replacement Therapy Treated with Intravenous Angiotensin II. Crit Care Med. 2018 Jun;46(6)
6. Nedel, W. L., Rech, T. H., Ribeiro, R. A., Pellegrini, J. A. S. & Moraes, R. B. Renal Outcomes of Vasopressin and Its Analogs in Distributive Shock. *Crit Care Med* **47**, e44–e51 (2019).
7. Hu, R. T. *et al.* Continuous bladder urinary oxygen tension as a new tool to monitor medullary oxygenation in the critically ill. *Crit Care* **26**, 389 (2022).
